# Access for Laparoendoscopic Single Site Surgery

**DOI:** 10.1155/2010/943091

**Published:** 2010-06-15

**Authors:** Sharona B. Ross, C. Whalen Clark, Connor A. Morton, Alexander S. Rosemurgy

**Affiliations:** Division of General Surgery, The Department of Surgery, Tampa General Hospital, Digestive Disorders Center, University of South Florida, Tampa, FL 33601, USA

## Abstract

Laparoscopic surgery is the standard of care for many abdominal and pelvic operations and is widely applied today. LESS (Laparo-Endoscopic Single Site) surgery, originally attempted in the 1990s, is an advanced minimally invasive approach that allows laparoscopic operations to be undertaken through a small (<15 mm) incision in the umbilicus, a preexisting scar. The presence of a preexisting scar allows LESS surgery to be essentially scarless, which is the key benefit to LESS operations. Herein, we review our experience with over 500 LESS operations and discuss the key techniques to establishing access to the peritoneal cavity. We review the options for obtaining access, available instrumentation, common challenges and solutions for access. We conclude that LESS surgery is safe and provides outcomes with superior cosmesis relative to conventional laparoscopy. LESS surgery should be embraced, as patient demand is rapidly increasing.

## 1. Introduction

Beginning in the late 1980s, conventional laparoscopic surgery has evolved to become the standard of care for many abdominal operations and widely utilized across the spectrum of abdominal and pelvic surgery. In 1992, the NIH consensus conference statement concluded that laparoscopic cholecystectomy was the standard of care in good risk patients [1]. Similarly laparoscopy has become the golden standard in colorectal surgery [2]. Single-site laparoscopic surgery was first attempted in the 1990's, but only in 2007 began as a concerted surgical approach. Single-site laparoscopic surgery is recognized by many different names and acronyms, all focusing on some aspect of its uniqueness. The acronyms have generally been trademarked to “protect” them or restrict their use. LESS (Laparo-Endoscopic Single Site) surgery is an acronym that was intentionally introduced into the public domain to avoid restriction of its application. LESS SCAR (Laparo-Endoscopic Single Site Surgery Consortium for Assessment and Research) became operational in 2008 to organize and direct application of single-site laparoscopy. The deliberations from that first meeting were far reaching and should help guide LESS surgery safely while it matures [3]. LESS SCAR met again in July 2009 and a report of that meeting is being generated.

LESS surgery is an advanced minimally invasive operative approach in which operations can be undertaken laparoscopically through a single small (i.e., 12–15 mm) incision, typically placed at the patient's umbilicus. Similar to conventional laparoscopic operations, LESS surgery is conventionally utilized under general anesthesia. Since the LESS operation is undertaken and completed through the umbilicus, a scar itself, the operation does not usually leave any visible “footprint.” In that regard, LESS surgery is “scarless.” The incision is made through an existing scar (i.e., the umbilicus) and, as a result, does not leave a new, or another scar. The theorized benefits behind this approach include less postoperative pain, faster recovery time, fewer complications, and better cosmetic outcomes. While we believe that LESS surgery results in a more rapid resumption of usual and functional activities, LESS Surgery is really all about “no scar.” Pain reduction and quicker return to functional activities will be hard to prove relative to conventional laparoscopy because conventional laparoscopy is well-tolerated.

Our experience with LESS Surgery began in the Fall of 2007. We initially undertook LESS cholecystectomy. It is an operation without too many “moving parts” and without reconstruction. We also undertooke a volume of cholecystectomies that we felt sufficient to allow for study and continuous procedural refinement. After initiating LESS cholecystectomy, we broadened our application of LESS surgery to include a host of operations commonly undertaken in our practice. To date, we have undertaken more than 500 LESS operations (Table 1). Others have reported experience with LESS donor nephrectomy, LESS bariatric procedures, and LESS pediatric procedures [4–8].

## 2. Ports and Trocars

As LESS surgery was being initially undertaken, access into the peritoneal cavity was achieved using existing commercially available ports, which used techniques and led to results we found lacking. As yet, access into the peritoneal cavity to achieve LESS surgery has not been standardized. Various methods have been described including.

**Figure 1:**
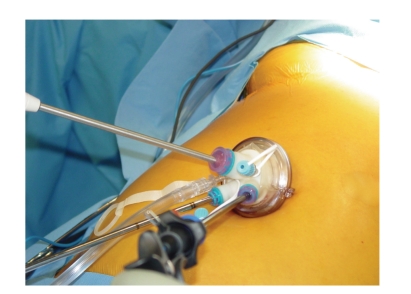
LESS TriPort.

**Figure 2:**
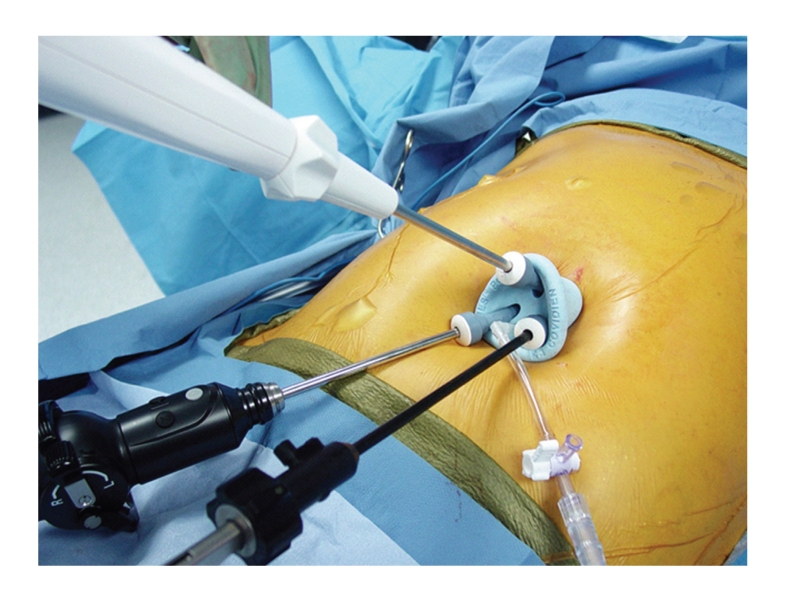
SILS port.

1. A single umbilical skin incision through which multiple individual trocars are placed through multiple fascial punctures, with skin flaps raised as necessary [9, 10].
2. A single umbilical skin incision through which a multitrocar port is inserted. There are a number of commercially available ports that fit this description, two of which are depicted in Figures 1 and 2:
  - the TriPort (Advanced Surgical Concepts, Wicklow, Ireland) (Figure 1)
  - the SILS port (Covidien, Norwalk, Conn) (Figure 2)
  - the Uni-X Single Port System (Pnavel Systems, Inc., Morganville, New Jersey)
  - the AnchorPort (Surgiquest Inc, Orange, CT)
  - the GelPort (Applied Medical, Rancho Santa Margarita, California)

**Figure 3:**
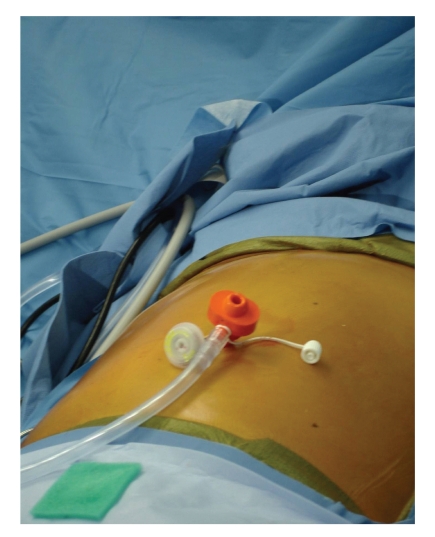
Two 5 mm trocars in place at the umbilicus.

**Figure 4:**
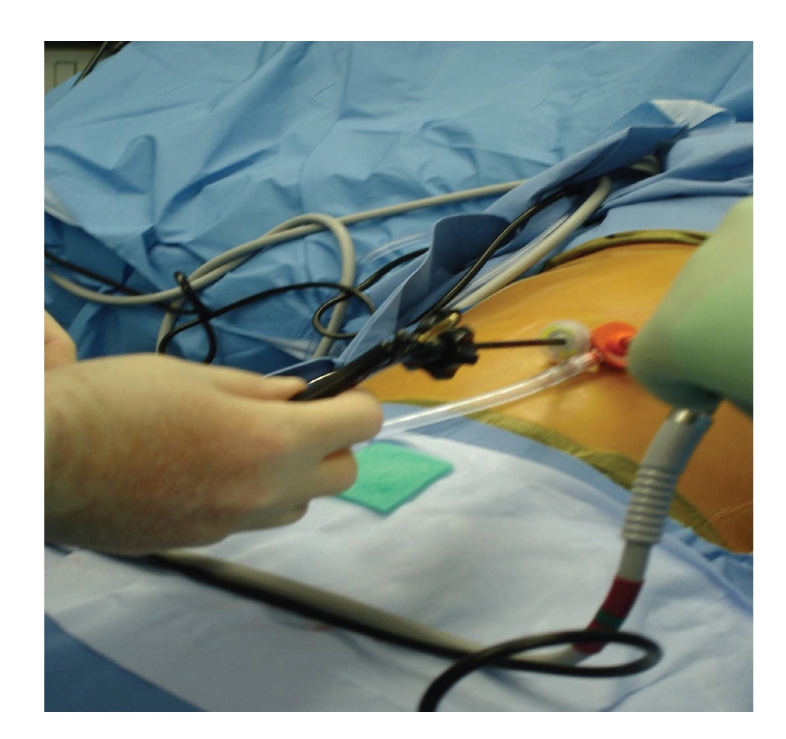
Instruments in two 5 mm trocars. Competition for space can be a problem, practically with a 5 mm scope that has a light source entering at 90 degrees, as shown.

Initially, we approached LESS surgery utilizing several individual trocars, the first approache is noted above. We would make a small incision at the umbilicus and insert a 5 mm trocar. Then, with pneumoperitoneum, we would insert additional trocars through the fascia adjacent to the first trocar placed (Figure 3). With this approach, air leaks were common and pneumoperitoneum was often lost. also, the heads on the trocars were too large and they “banged” into each other, interfering with instrument manipulation and operation of the laparoscope (Figure 4). These trocars contributed to too much clutter at the umbilicus. Today, we recommend using this approach for LESS surgery only when undertaking LESS cholecystectomy and then we use only two 5 mm ports. This approach requires the use of adjunctive sutures for retraction and displacement to allow for “puppeteering” of the gallbladder. The first of these sutures is placed through the abdominal wall along the midclavicular line, into the fundus of the gallbladder, and out the abdominal wall near where the suture first entered the abdomen. Then, a second suture is placed through the abdominal wall near the xyphoid process to the right of the falciform ligament, through the infundibulum of the gallbladder, and then back out through the right abdominal wall along the anterior axillary line. These sutures allow retraction on the gallbladder and facilitate cholecystectomy. Acceptable outcomes were achieved in our first one hundred patients using this technique and others have begun to embrace a similar technique [11–13].

The multitrocar ports are thought to require larger incisions at the umbilicus (i.e., at the skin), but only a single fascial incision for passage of multiple instruments and a laparoscope. Our experience is primarily with the first two multi-trocar ports listed. We believe that these trocars do not require larger, meaning excessively large, incisions. The TriPort (Figure 1) has three access sites in addition to two sites for CO_2_ insufflation. It is easy to place because it has a very handy insertion device (i.e. an introducer) which allows the port to be easily extruded into position in the umbilicus or other single-incisions. The valves at each access site in the TriPort are fragile. Each valve, and the instruments used, should be lubricated with mineral oil to facilitate instrument placement across the valves and down the trocars. Mineral oil works better than water soluble gel because it does not “dry out.” A modification of this port allows four access sites for instruments, the QuadPort. The SILS port (Figure 2) has been used in a significant number of operations by us. It is harder to place than the TriPort, but it allows four instruments to be placed, including the laparoscope, if the insufflation trocar is removed and replaced by a 5 mm trocar with insufflation capabilities. Although, with this port the skin incision might be a bit longer than with the TriPort, but the umbilical ring usually remains intact and lack of scarring can be impressive. We have had limited exposure to the other multitrocar ports listed.

The incremental cost of the multiport trocars has received considerable attention. Cost of care today is an issue that cannot be ignored. However, the cost of such a port equates to about 6–8 minutes of operating room time, and they certainly save more than that by allowing the procedures to proceed more expeditiously. These ports are essential, we feel, in undertaking complex LESS procedures because they help avoid clutter and competition for space at the umbilicus. These ports can result in a very pleasing scar if care is taken with the incision. The ring of skin about the umbilicus cannot be violated.

## 3. How Access Is Obtained

**Figure 5:**
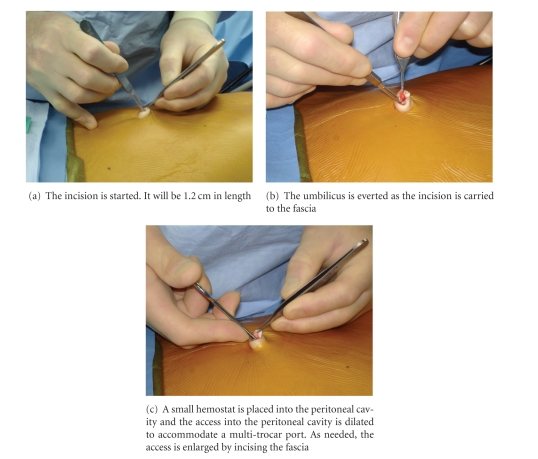
Obtaining access into the peritoneal cavity by direct cutdown inside the umbilical ring.

Once patients are intubated under general anesthesia they are prepped and draped in a sterile fashion, and local anesthesia is injected into the umbilicus. We use commercially available marcaine mixed with epinephrine. An approximately 1.2 cm long vertical incision is made in the umbilicus with great caution not to cut the umbilical ring, or the skin ring around the umbilicus (Figure 5(a)). Dividing the umbilical ring will result in a permanent deformity of the umbilicus. As the incision is made, the umbilicus is everted (Figure 5(b)). A small fascial defect is frequently, almost always, present at the base of the umbilicus and can be gently dilated to allow placement of a TriPort or a SILS port with copious amount of lubrication (Figure 5(c)). Figure 5 demonstrates the sequence of maneuvers used to obtain access. We use water soluble gel (e.g., KY jelly or Lubifax) to facilitate placement of the multitrocar ports. The TriPort provides two 5 mm trocars, one 12 mm trocar, and two conduits for CO_2_ insufflation. The SILS port provides for three 5 mm trocars, one of which can be upsized to 12 mm, as well as one conduit for CO_2_ insufflation. When we use the SILS port, the CO_2_ insufflation conduit is usually replaced by a 5 mm trocar with CO_2_ insufflation capabilities. This allows us to gain an extra (i.e., fourth) working trocar. When we use the 12 mm trocar, we use a reusable metal trocar with a rubber stopper for a valve. This consumes less space than commercially available trocars or the 12 mm trocar that accompanies the port. Once pneumoperitoneum is established, the operation begins with the insertion of a 5 mm deflectable tip laparoscope and 5 mm instruments. The instruments used vary according to the specific operation undertaken, while the laparoscope is always the same. A deflectable tip laparoscope is very important. 

Potential access problems or limitations one may encounter during LESS surgery include the following.

- Small umbilical ring—this will limit the size of the incision before the umbilical ring is disfigured. When making an incision in a small umbilicus, extend the incision in a cruciate manner to avoid cutting the umbilical ring.
- High BMI with a thick abdominal wall—this makes the path across the abdominal wall long and may limit across the abdominal wall with some of the ports, such as the SILS port.
- Adhesions from previous operations—these may limit the access to the peritoneal cavity once the incision has been made.
- Port availability—not every hospital has commercially available multi-trocar ports available.
- Lack of instrument triangulation—this is a general issue with LESS surgery. This is reduced with use of an articulating laparoscope. With a deflectable tip laparoscope, we have not found this to be a problem. We generally do not use articulating instruments.
- Instruments are too short—it is difficult to reach all areas of the peritoneal cavity from the umbilicus in a taller patient. How “tall” is “tall”? In part that depends on what operation is being undertaken. For example, reducing a large hiatal hernia can be impossible in a tall patient because the instruments are just not long enough.
- Inadequate imaging—this is resolved by using a deflectable tip laparoscope with a bright light source and high quality imaging.
- Incision too small for specimen extraction—this is not uncommon for any operation beyond cholecystectomy. The TriPort aids in specimen extraction because the top of the port can be “popped off” and the specimen removed. Then the top can be easily replaced on the port and pneumoperitoneum can be re-established. With the SILS port, tissue extraction requires removal through a trocar as large as 12 mm or removal of the SILS port. Then to re-establish pneumoperitoneum, the SILS port has to be placed as in the beginning of the operation. Therefore, we often leave tissue extraction to the last step in the operation prior to wound closure.
- Insufflation leak (CO_2_ insufflation leak) —this is a *BIG* problem when LESS surgery is undertaken through multiple individual trocars. This is less of an issue with the ports that provide multiple access points such as the TriPort or the SILS port. If an insufflation leak occurs, add a second insufflator.
- Risk of hernia—this is talked about a lot. Hernias at the umbilical incision are not much of an issue with conventional laparoscopy which may involve as much as a 12 mm incision for a large Hasson trocar. LESS surgery involves a 12–15 mm incision, and hernias with LESS surgery should not develop at a higher rate. Hernias at the umbilical incision have not been an issue in our practice.

Occasionally patients may present with a very small umbilical ring, which limits the size of the umbilical incision and therefore the access opening. In this instance the vertical incision should be enlarged transversally (in a cruciate manner) within the umbilicus. The incision may be quite cruciate. To ensure no visible scar at the completion of the operation, avoid cutting the umbilical ring. 

Patients with high BMI represent a very challenging group of patients for single incision laparoscopic operations. Patients with BMI less than 26–28 kg/m^2^ are considered the ideal candidates for this surgical approach. Patients with BMI greater than 30 have a higher risk of having large amount of intraperitoneal fat, which makes exposure more difficult and/or having a thick abdominal wall which limits the use of LESS ports. The TriPort is preferred in patients with a thick abdominal wall because the sleeve mechanism of the TriPort allows it to transverse greater abdominal wall thickness.

Patients with history of previous “open” operations represent another challenging group of patients. Patients with previous abdominal incisions tend to have extensive intraperitoneal adhesions that potentially make any laparoscopic operation difficult. However, a history of prior abdominal operation(s) should not discourage surgeons from attempting a LESS approach. Instead it should alarm the laparoscopic surgeon that it may be a difficult, especially to get started, but not an impossible mission. “What kind of operation(s)”, “with what kind of complication(s)”, and “undertaken how long ago” will be issues. As long as safety can be ensured, the LESS surgery approach can be attempted and hopefully completed.

LESS surgery can be undertaken with standard laparoscopic instrumentation with some limitations. The freedom of the hands is relatively restricted, which leads to clashing and “banging” of instruments, and the fixed port at the umbilicus potentially creates a long distance to the surgical site requiring longer instruments. A deflectable tip allows the laparoscope to be placed to the side, getting it out of the way. Currently there are many new innovative laparoscopic instruments that aid with the single incision application. Specialized articulating instruments make LESS Surgery easier and simpler for some, possibly many. It has been our choice to generally avoid articulating instruments, though we use some reticulating instruments. We generally find articulating instruments to be superfluous. The articulating instruments provide a degree of triangulation, but the wrist motion consumes the space outside the peritoneal cavity and often proves unwieldy. In addition, the lack of long instruments presents new challenges particularly with tall patients. In our experience, instruments designed for Bariatric Surgery are longer and help overcome length limitations. As new instruments continue to develop to accommodate the new paradigm of LESS Surgery, it is likely that technical difficulties as mentioned above will be minimized.

LESS Surgery should only be undertaken with the appropriate imaging technology. For basic laparoscopic procedures, such as cholecystectomy and appendectomy, a standard 5 mm laparoscope provides sufficient visualization of the surgical site. However, for other procedures, the length and visibility provided by the standard laparoscope present some limitations. First, the light source for the scope enters at 90° to the scope. This adds clutter to the area about the umbilicus and to the operative field. Second, the distance from the umbilicus to the surgical site may be longer than with conventional laparoscopic approaches for instance, Nissen fundoplication or Heller myotomy requires close proximity to the esophageal hiatus and therefore a longer scope for adequate visualization. Third, since instruments and scopes enter the abdominal cavity via the same fascial opening, a deflectable tip laparoscope is advantageous in allowing surgeons to clearly view the operative field and create a sense of triangulation. EndoEye laparoscope (Olympus Surgical & Industrial America Inc, Center Valley, PA) allows panoramic view of the surgical site with minimal movements by the laparoscope operator. The dials on the scope allow the operator to manipulate the tip of the laparoscope with the shaft off line and out of the way while keeping the scope flat/parallel to the patient's body and providing the working ports with a higher degree of freedom.The fact that the laparoscope can be removed from the area about the umbilicus by laying it flat/parallel to the patient's body is very helpful in the conduct of the LESS operation.

The size of the umbilical incision used in LESS Surgery potentially limits the extraction of large intra-abdominal organs, tumors, or specimens. As mentioned above, there are differences as to how the various different ports facilitate or hinder specimen extraction. If a SILS port is used, make sure that the operation other than specimen extraction has been completed. Then when the port is removed to extract the specimen, the port will not have to be placed again, and closure of the umbilicus can begin.

If LESS surgery is undertaken with multiple individual trocars, CO_2_ leakage around the trocars is common because as torque is placed on the trocars the holes in the fascia enlarge. When this happens, the first step should be to add another insufflator. Putting gauze or materials about the trocars to prevent CO_2_ leakage is a waste of time. There is no ready access to place sutures around the trocars. Both the TriPort and SILS port have separate designated insufflation conduits. The TriPort has two built-in insufflation ports while the SILS port has one. One is almost always enough. When we use the SILS port and experience CO_2_ leaks, we remove one of the low profile 5 mm trocar and exchange it with another low profile trocar that has built-in insufflation access. This exchange allows us to overcome the air leak and proceed with the operation safely.

**Figure 6:**
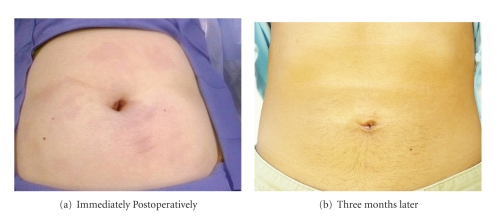
The Postoperative Scarless Umbilicus.

Last but not least, many surgeons fear that making a 12 to 15 mm incision at the umbilicus increases the risk of hernia. In our experience the rate of incisional hernia has not increased beyond what we saw with conventional laparoscopy. We recommend approximately 12–15 mm vertical incision at the umbilicus and we repair this fascial defect with an absorbable suture in a figure-of-eight fashion. To date, we have done more than 500 LESS operations and had only one umbilical hernia. The hernia occurred during our initial experience with LESS Surgery when we repaired the umbilical fascial defect with one simple interrupted suture placed not in a figure-of-eight fashion. Figure 6 shows the typical cosmetic result and postoperative appearance of the umbilicus.

LESS surgery is here and now. It is our experience that LESS surgery can be completed safely with equivalent outcomes to conventional laparoscopy. There does exist a learning curve with LESS surgery. Patients are going to demand LESS surgery and laparoscopic surgeons will have to embrace their demands.

## Figures and Tables

**Table 1:** Operations undertaken by our institution utilizing the LESS approach.

(i)	Giant hiatal hernia repair, with Nissen/Toupet fundoplication
(ii)	Heller myotomy and anterior fundoplication
(iii)	Splenectomy
(iv)	Distal pancreatectomy/removal pancreatic tumor
(v)	Adrenalectomy
(vi)	Inguinal hernia
(vii)	Hepatic cystectomy
(viii)	Salpingo-opherectomy
(ix)	Mesenteric mass excision
(x)	Appendectomy
(xi)	Resection of gastric/small bowel tumors (i.e. GIST tumor)
(xii)	Right colectomy
(xiii)	Low Anterior Resection (LAR)
(xiv)	Cholecystectomy ± cholangiogram
(xv)	Combined operations: Nissen/Toupet fundoplication and cholecystectomy ± cholangiogram
	Heller myotomy and anterior fundoplication and cholecystectomy Hysterectomy and cholecystectomy
